# Accurate Interpretation of SARS-CoV-2 Antigen Detection by Immunochromatography

**DOI:** 10.3389/fmed.2022.949554

**Published:** 2022-06-29

**Authors:** Wenxia Shao

**Affiliations:** Department of Clinical Laboratory, Affiliated Hangzhou First People's Hospital, Zhejiang University School of Medicine, Hangzhou, China

**Keywords:** COVID-19, SARS-CoV-2, immunochromatography, antigen, false positive, false negative

## Abstract

SARS-CoV-2 is a serious infectious respiratory virus that can cause lung, heart, kidney, and liver damage and even cause death. Early diagnosis of SARS-CoV-2 infection is vital for epidemic prevention and control. At present, the gold standard of COVID-19 diagnosis is nucleic acid detection of SARS-CoV-2. However, the nucleic acid detection of SARS-CoV-2 requires high site requirements and technology requirements, and the detection is time-consuming and cannot fully meet clinical needs. Although SARS-CoV-2 antigen test results cannot be directly used to diagnose COVID-19, positive results can be used for the early triage and rapid management of suspected populations. However, due to the limitations of the methodology itself, the SARS-CoV-2 antigen test is prone to produce false-positive and false-negative results in the process of detection. It is urgent to develop a batch of SARS-CoV-2 antigen reagents based on new detection technology and detection principles to overcome the defects of existing technologies.

## Introduction

SARS-CoV-2 is a severe infectious respiratory virus that can cause injury to the lung, heart, kidney, and liver and even cause death (1–9). It is well-known that the basic principles of infectious disease control are controlling the source of infection, cutting off the route of transmission, and protecting the susceptible population. However, the existing vaccines and monoclonal antibodies are less effective because the virus mutates so quickly that immune escape is severe (10–15). In view of this situation, it is difficult to curb the COVID-19 epidemic solely from the perspective of patient prevention and treatment. It is necessary to control the source of infection further and cut off the transmission route of SARS-CoV-2 to control the epidemic situation thoroughly. To achieve these goals, timely, and accurate diagnosis and identification of COVID-19 patients are critical. At present, the gold standard of COVID-19 diagnosis is nucleic acid detection of SARS-CoV-2 (16, 17). However, the nucleic acid detection of SARS-CoV-2 requires professionals to perform in a PCR laboratory with level II biosafety (18, 19). Because of its high skill requirements and site requirements, it cannot be widely promoted, especially in some primary medical institutions. In addition, due to the complexity of the operation, standard SARS-CoV-2 nucleic acid detection takes 4–6 h to complete. Moreover, the report will be longer for limited fluxes and large specimens. This may lead to delays in identifying COVID-19 patients in medical institutions. The simultaneous gathering of COVID-19 patients and non-COVID-19 patients in health care facilities not only increases the risk of cross-infection but also occupies a large number of medical resources, putting great pressure on the prevention and control of COVID-19 in medical institutions.

**Figure 1:**
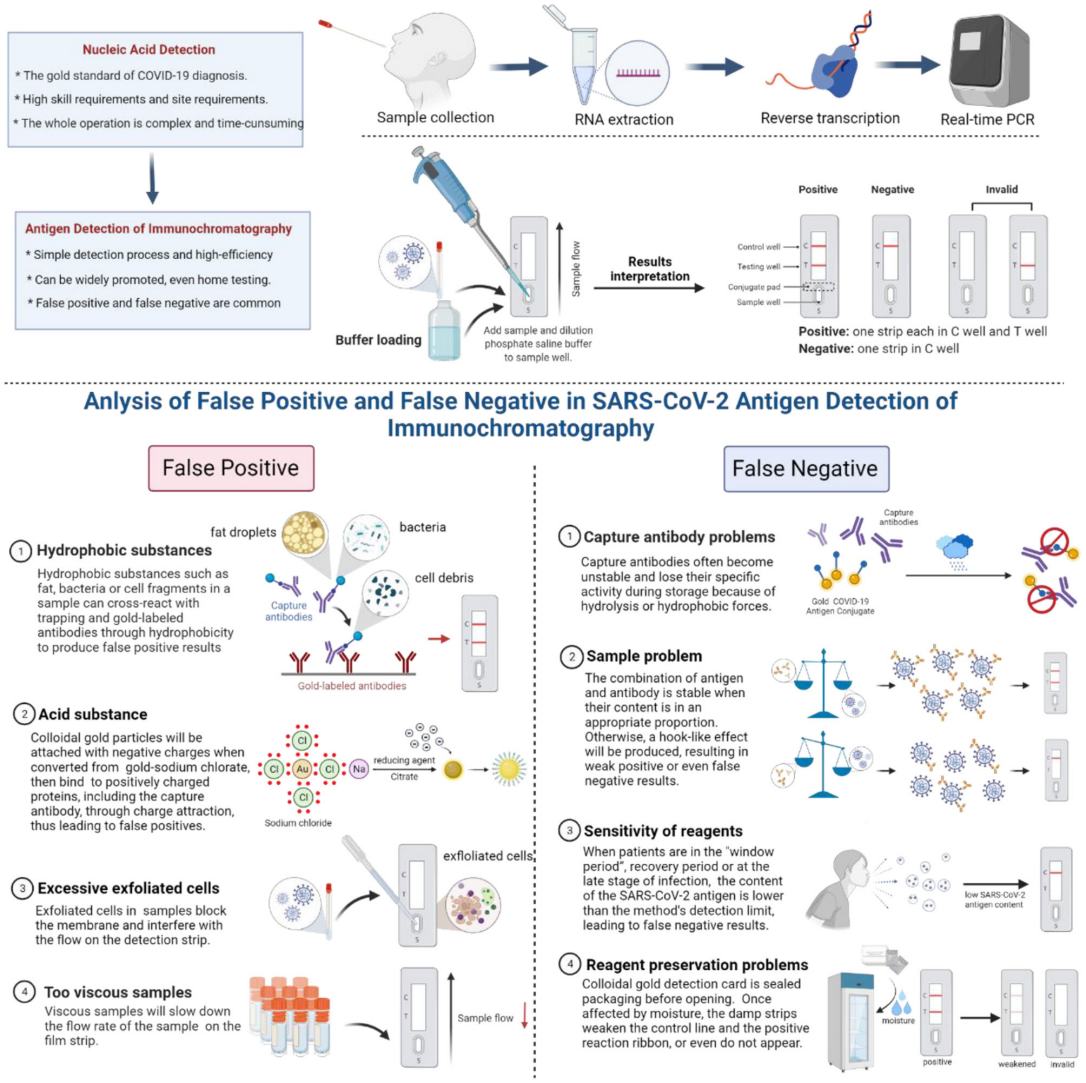
Common causes of false positives and false negatives of colloidal gold immunochromatography.

Colloidal gold immunochromatography is a rapid and straightforward method for detecting SARS-CoV-2 antigen. However, due to the factors of reagents and samples, some false positive or false negative results were caused (20, 21). This paper will analyze the common causes of false positives and false negatives in using the colloidal gold immunochromatography SARS-CoV-2 antigen assay to provide a reference for the interpretation of the results (Figure 1).

## Common Causes of False Positives of Colloidal Gold Immunochromatography

### Hydrophobic Substances

Proteins rich in non-polar amino acids, such as tryptophan, valine, leucine, isoleucine, or phenylalanine, will have a strong binding effect through hydrophobic forces once they are very close to each other (within a distance of <1 nm). Therefore, hydrophobic substances such as fat, bacteria, or cell fragments in a sample can cross-react with trapping and gold-labeled antibodies through hydrophobicity to produce false-positive results. These non-specific bonds can be decomposed by adding surfactants or hydrophilic polymers.

### Acid Substance

When sodium chlorate is converted into colloidal gold, the reducing agent citrate is used with a layer of negative charge attached to the surface of colloidal gold particles, which gives the colloidal gold particles a negative charge. When the pH of the sample is less than the isoelectric point of zwitterions, the protonation of zwitterions is positively charged. In particular, protein regions rich in lysine and arginine are positively charged below the isoelectric point of lysine (pH 10.4) and arginine (pH 12.5). In this way, the negatively charged colloidal gold can bind non-specifically to positively charged proteins, including the capture antibody, through charge attraction, thus leading to false positives.

### Excessive Exfoliated Cells

Some samples may contain a large number of exfoliated cells, which have the potential to block the membrane and interfere with the flow of gold standard solution on the detection strip.

### Too Viscous Samples

Some samples may be very viscous, which will slow down the flow rate of the sample and sample diluent on the film strip. When there are not enough samples and sample dilutions in the detection system to move the gold-labeled antibody along the detection band, the colloidal gold particles will also adhere to the capture antibody band.

### Reagent Problem

It is generally recommended that the reagent should be used immediately after unsealing, and the exposure time should not be too long. If the exposure time is too long, the reagent will deteriorate, and false-positive results will appear.

## Common Causes of False Negatives of Colloidal Gold Immunochromatography

### Problems of Capture Antibodies

Capture antibodies often become unstable and lose their specific activity during storage, which may be due to the destruction of hydrolysis due to a wet environment and insufficient drying or the destruction of hydrophobic forces. The former requires that reagents be stored in a dry environment, and the detection should be carried out in a controlled dry environment. The latter may be the characteristic of the antibody itself, which can be solved by replacing the antibody or removing the capture antibody layer and then treating the membrane with a surfactant.

### Sample Problem

The combination of antigen and corresponding antibody is the most stable when the content of antigen and corresponding antibody is in a certain appropriate proportion during the detection process. If the concentration of SARS-CoV-2 antigen is too high, a hook-like effect will be produced, making the ratio of antigen and antibody imbalanced and resulting in weak positive or even false-negative results. In particular, the hook effect is more likely to occur in the detection based on the one-step principle. At this point, more accurate results can be obtained if the sample is diluted properly.

### Sensitivity of Reagents

One of the main reasons for the false-negative detection of SARS-CoV-2 antigen by the colloidal gold immunochromatography is the limited sensitivity of the reagent itself. When the content of the SARS-CoV-2 antigen is lower than the method's detection limit, false negatives may occur. Therefore, patients are prone to have false-negative results in the “window period” with low SARS-CoV-2 antigen content, in the recovery period, or at the late stage of infection (22–25).

### Reagent Preservation Problems

Colloidal gold detection card is sealed packaging before opening. It is easily affected by moisture when the detection card is stored in a 4°C refrigerator after opening. The damp strips weaken the control line, and the positive reaction ribbon or even do not appear. Therefore, the sensitivity of the detection will decrease or be invalid, affecting the accuracy of the test results.

### Immune Escape

The SARS-CoV-2 mutant reduces the detection rate of the SARS-CoV-2 antigen reagent, especially the vaccine escaping the SARS-CoV-2 mutant (26–29).

## Summary and Prospects

The immunochromatographic colloidal gold method has the advantages of rapid and straightforward operation. Nevertheless, due to the limitation of the methodology itself, it is easy to produce false positive and false negative results in the application process. It is urgent to develop a batch of SARS-CoV-2 antigen reagents based on new detection technology and detection principles to overcome the defects of existing technologies.

## Author Contributions

WS contributed to the content, writing, and critical review of the manuscript.

## Conflict of Interest

The author declares that the research was conducted in the absence of any commercial or financial relationships that could be construed as a potential conflict of interest.

## Publisher's Note

All claims expressed in this article are solely those of the authors and do not necessarily represent those of their affiliated organizations, or those of the publisher, the editors and the reviewers. Any product that may be evaluated in this article, or claim that may be made by its manufacturer, is not guaranteed or endorsed by the publisher.
